# Maternal and Perinatal Outcome in Patients With Eclampsia: A Study Done at a Tertiary Care Centre

**DOI:** 10.7759/cureus.45971

**Published:** 2023-09-26

**Authors:** Pratibha Dixit, Tarunendra K Mishra, Devendra Nargawe, Sandeep Singh

**Affiliations:** 1 Department of Obstetrics and Gynaecology, Government Medical College and Hospital, Ratlam, IND; 2 Department of General Medicine, Government Medical College and Hospital, Ratlam, IND; 3 Department of Pediatrics, Government Medical College and Hospital, Ratlam, IND; 4 Department of General Medicine, Netaji Subhash Chandra Bose Medical College, Jabalpur, IND

**Keywords:** preterm delivery, hypertension, parity, maternal outcomes, fetal outcomes, eclampsia

## Abstract

Background

One of the leading causes contributing to morbidity and mortality globally is attributed to eclampsia. Hence, it is vital to comprehensively review each female having eclampsia and to evaluate the factors that govern the outcomes in females with eclampsia.

Aim

To decode the fetal and maternal outcomes in subjects having eclampsia and to evaluate various factors that govern the outcomes.

Methods

This retrospective cohort and epidemiological study commenced at the Department of Obstetrics and Gynaecology, Netaji Subhash Chandra Bose Medical College, Jabalpur, Madhya Pradesh, in January 2016 till April 2017, and included females that either developed eclampsia in hospital stay duration or presented with pre-existing eclampsia. In included females, various fetal and maternal parameters were assessed along with the outcome of pregnancy. The institutional data records and the database were also used to determine the prevalence and incidence of eclampsia. Baseline maternal parameters were recorded from the already-existing institute data. These included the gestational age (in years), socioeconomic status, educational attainment, parity, gravidity, and the number of weeks of gestation present at the time of delivery. Antenatal care data assessed were blood pressure recordings, any proteinuria documented in the data, and the number of antenatal visits by the subjects. Statistical analysis was performed to assess both parameters.

Results

In the current investigation, there were 0.34% eclampsia cases among females visiting the institution for deliveries. Incidences of stillbirth were seen in 19.04% and 8% of study participants, respectively. We found 9.52% (n=4) of female infants to have perished from eclampsia. Preterm births, a delayed start to the treatment, and insufficient care were all linked to poor foetal and mother outcomes. The longer the period between the beginning of a fit and delivery, the greater the likelihood of unfavourable results. Seizure onset before or after birth, parity, or subject age had no impact on mother or foetal health. The p-value for statistical significance was kept at 0.05.

Conclusion

Most of the research participant women, had intrapartum eclampsia, postpartum eclampsia, and antepartum eclampsia, based on the time of the convulsions in relation to the labor. It was highlighted that there was no conclusive evidence linking the date of the fit's beginning to unfavourable results or an elevated risk of complications. Neonatal mortality and stillbirth were observed with vaginal delivery in eclampsia cases. Outcomes in eclampsia can be improved by early treatment initiation, timely and appropriate referral, early disease recognition, and appropriate antenatal care.

## Introduction

In Asia and Africa, only hypertensive disorders of prevalence in pregnancy account for close to 10% of all maternal deaths. Eclampsia is a condition characterized by high blood pressure, headaches, blurry vision, and convulsions. Eclampsia and pre-eclampsia are regarded as significant contributors to neonatal and maternal mortality and morbidity when all conditions that complicate and adversely influence pregnancy are taken into account. Eclampsia is the third most frequent cause of maternal mortality among direct obstetric factors [1].

Maternal death in eclampsia is seen secondary to the factors that are preventable, including PPH (postpartum haemorrhage), APH (antepartum haemorrhage), pulmonary oedema, aspiration pneumonia, coagulation failure, ARF (acute renal failure), and/or cerebrovascular haemorrhage. Compared to affluent countries, where perinatal mortality rates range from 5% to 11%, perinatal mortality rates in poor countries are much higher, with approximately 40% of recorded deaths. The maternal mortality amongst cases of eclampsia was 31.8% and perinatal loss was 38.6% [2,3]. 

Eclampsia is a condition characterized by high blood pressure, headaches, blurry vision and convulsions, and in preeclampsia, the mother’s high blood pressure reduces the blood supply to the fetus, which may get less oxygen and fewer nutrients. Eclampsia is mostly diagnosed with the early detection of preeclampsia because there are no reliable clinical symptoms or laboratory tests for the prediction of its development. Hypertension is regarded as a defining symptom of eclampsia when making a diagnosis. Preeclampsia, on the other hand, typically has an insidious beginning, with symptoms typically appearing in the latter stages of the condition, whereas pathological abnormalities are visible [4].

In nearly 16% of the eclampsia cases, hypertension may not be reported at all. It has been reported that proteinuria might be absent in nearly 14% of the eclampsia cases and substantial proteinuria might be present in nearly half of the cases [5].

The easy availability and accessibility of healthcare facilities and centres to nearly all pregnant women can help in the timely detection and prevention of eclampsia and further maternal and perinatal mortality and morbidity in the affected subjects, which has been linked to a decrease in the incidence of eclampsia in developing countries [6].

The ubiquitous availability of prenatal care is still insufficient and inappropriate in underdeveloped nations. To prevent the mortality and morbidities brought on by eclampsia and pre-eclampsia, it is essential to give female patients with those conditions effective and prompt care [7]. 

With delivery the only current cure, preeclampsia also contributes significantly to prematurity, neonatal morbidity, and perinatal mortality. The early and ongoing efforts to examine and monitor the course of therapy and to assess the numerous factors that may affect the outcomes of eclampsia are significantly responsible for the prevention and appropriate management of eclampsia and pre-eclampsia [8].

The aim of this study was to examine the maternal and perinatal outcomes in women with eclampsia and to identify the many factors that may have an impact so that preventive actions may be taken to lessen the likelihood of negative outcomes.

## Materials and methods

The current retrospective epidemiological study was carried out at Netaji Subhash Chandra Bose Medical College in Jabalpur, Madhya Pradesh, India, from January 2016 to April 2017, after approval from the Institutional Ethical Committee (MPICE/2017/1913). Before the study began, verbal and written informed consent was obtained. Participants in the study were from the institute's Department of Obstetrics and Gynaecology.

Females who either acquired eclampsia during their hospital stay or who presented to the institution with eclampsia and gave consent were included in the research. Blood pressure ≥ 140 mmHg systolic or ≥ 90 mmHg diastolic on two separate readings taken at least four hours apart. Subjects with previously normal blood pressure and having proteinuria greater than or equal to 300 mg per 24-hour urine collection were included in the study. Women with known hypertension, renal disease, serious medical conditions, and who couldn’t give consent were excluded from the study. 

The research population consisted of 42 females who received pre-diagnosed eclampsia at the institute throughout the course of the investigation. The research population also included all neonates (n=42) born to eclamptic women or those who were hospitalized at the hospital after delivery and were identified as having postpartum eclampsia. The institutional data records and the database were also used as the base of our study to determine the prevalence and incidence of eclampsia. Prevalence is not basically measured, but in epidemiologic studies, we can measure it. Baseline maternal parameters were recorded from the already-existing institute data. These included the gestational age (in years), socioeconomic status, educational attainment, parity, gravidity, and the number of weeks of gestation present at the time of delivery. The data was collected from registers of the hospital record room. All cases who came for delivery services consecutively to the hospitals were included until the required sample size was fulfilled. This process took one month time. 

The study also gathered information on prenatal and postnatal care, the overall number of seizures (postpartum or intrapartum), the timing of the delivery, including whether it occurred postpartum, intrapartum, or antepartum, treatment given to the subject prior to a referral from the peripheral centres, and the time it took the subject to arrive at the proper medical facilities. The time between delivery and the commencement of a fit, the length of time it took to start the proper therapy, an eclamptic episode, the presence or absence of proteinuria as determined by the dipstick technique at the time of admission, and blood pressure at that time. The subject's file, the prenatal cards, and the referral paperwork were all used to gather the data. 

Lactate dehydrogenase, alanine aminotransferase, serum aspartate aminotransferase, serum bilirubin, blood urea, serum creatinine, and total blood count were the laboratory values evaluated in the research patients. Blood pressure readings, any proteinuria noted in the data, and the number of prenatal visits made by the individuals were all evaluated as part of the antenatal care data. The World Health Organization (WHO) criteria, which recommend a model of at least four visits, were used to evaluate the prenatal care provided to study participants. 

The criteria assessed whether any anticonvulsants had been given to the subject during the eclamptic episode prior to the subject's referral, previous records sent, records of the treatment provided, and whether the medical support system was sent along with the female during their referrals, as well as the data sent along with the females, in order to determine whether the referral was accurate.

Maternal complications such as mortality rate, brain haemorrhage, transient cortical blindness, posterior reversible leukoencephalopathy (PRES), need for ventilation, septicemia, pulmonary oedema, acute renal failure, HELLP (haemolysis, elevated liver enzymes, low platelet count) syndrome, PPH, and APH in subjects with eclampsia were assessed in the outcomes study.

Low birth weight, defined as a birth weight of 200 grams or less to 2499 grams, and stillbirth, defined as a birth weight of 500 grams or less in a newly born neonate with no evidence of life, were the fetal outcomes evaluated in the current study. The incidence of low birth weight, neonatal outcomes, and stillbirth rate were the key fetal outcomes evaluated.

Statistical analysis

The SPSS program version 21.0 (IBM Corp., Armonk, USA) was used to statistically test the study's data using the t-test and Chi-square test. The frequency, percentage, mean, and standard deviation of the data were all expressed. The p-value for statistical significance was kept at 0.05.

## Results

In the current study, there were 42 females diagnosed with eclampsia out of the 12,969 females giving birth at the institute over the specified study period. Concerning the maternal outcomes, it was seen that vaginal and cesarean delivery was done in 20 and 22 females, respectively. In 10% (n=2) and 9.09% (n=2) females, death was reported in the vaginal and cesarean delivery groups, respectively, which was non-significant with p=0.283. Brain hemorrhage was seen in 4.54% (n=1) of subjects from the cesarean delivery group (p=0.422). Transient cortical blindness as a maternal complication was reported in 5% (n=1) and 4.54% (n=1) subjects each from the vaginal and cesarean delivery group respectively (p=0.316).

Ventilation was required by 15% (n=3) and 9.09% (n=2) of the females in the groups of vaginal and caesarean births, respectively, with a p-value of 0.243. Postpartum haemorrhage was observed in 15% (n=3) and 4.54% (n=1) subjects from the vaginal and caesarean delivery groups, respectively (p=0.332). Antepartum haemorrhage was reported in 5% (n=1) and 4.54% (n=1) subjects from the vaginal and caesarean delivery groups, respectively (p=0.176) and DIC (disseminated intravascular coagulation) was observed in 5% (n=1) subjects from the vaginal delivery group. According to Table 1, there was no statistically significant difference between the vaginal delivery group and caesarean delivery group for septicemia, pulmonary oedema, acute renal failure, and HELLP syndrome, with corresponding p-values of 0.750, 0.134, 0.983, and 0.751. Subjects with premonitory symptoms and signs were found with a borderline elevated risk of pulmonary oedema but no other serious complications.

**Table 1:** Maternal outcomes in the study subjects

Complications	Vaginal delivery		Caesarean delivery		p-value
	Number (n=20)	Percentage (%)	Number (n=22)	Percentage (%)	
Death	2	10	2	9.09	0.283
Brain hemorrhage	0		1	4.54	0.422
Transient cortical blindness	1	5	1	4.54	0.316
Required ventilation	3	15	2	9.09	0.243
Posterior Reversible Leukoencephalopathy	5	25	2	9.09	0.641
Postpartum hemorrhage	3	15	1	4.54	0.332
Antepartum hemorrhage	1	5	1	4.54	0.176
Disseminated intravascular coagulation	1	5	0		0.807
Septicemia	1	5	1	4.54	0.750
Pulmonary edema	2	10	4	18.1	0.134
Acute renal failure	2	10	3	13.63	0.983
HELLP syndrome	4	20	2	9.09	0.751

One woman had a seizure while she was in the hospital; the other women either reported having convulsions before going to the hospital or were referred from other outlying sites. The rate of appropriate referral among the referred females was relatively low, with 7% of respondents only receiving an adequate referral. Compared to females who were referred after receiving therapy and oxygen supplementation, the group of females where sufficient referral was not performed had a higher risk of developing DIC, a higher death rate, and a higher requirement for mechanical ventilation.

Among the 42 research participants, 2.38% (n=1), 14.28% (n=6), and 73.80% (n=31) of the women had intrapartum eclampsia, postpartum eclampsia, and antepartum eclampsia, respectively, based on the time of the convulsions in relation to the labor pain. It was highlighted that there was no conclusive evidence linking the date of the fit's beginning to unfavourable results or an elevated risk of complications. All of the female patients with eclampsia who were admitted to the facility had blood tests done. 14.28% (n=6) and 16.66% (n=7) of the study's female participants showed abnormal liver function tests (LFT) and elevated blood creatinine levels, respectively. Before the gestational age of fewer than 37 weeks, 52.38% (n=22) of females reported having pre-eclampsia. In these subjects, the neonate was at higher risk of developing complications, including the need for admission to the nursery and intrauterine growth retardation (IUGR).

In these subjects, the neonate was at higher risk of developing complications, including the need for admission to the nursery and intrauterine growth retardation (IUGR). However, no major increased risk was seen in the mother of this fetus. One significant result of interest from the current study was that only 14.28% (n=6) of the participants were able to go to a medical facility within the first six hours of the convulsion beginning, and only 21.42% (n=9) of the subjects were able to get to the hospital within the first 24 hours. When they reported, the majority of these women had disseminated intravascular coagulation and pulmonary oedema. When compared to participants who arrived at the healthcare facility within six hours or acquired eclampsia while in the hospital, these subjects had a higher chance of death, as shown in Table 2.

**Table 2:** Fetal outcomes in the study

Parameters	Vaginal delivery		Cesarean delivery		p-value
	Number (n=20)	Percentage (%)	Number (n=22)	Percentage (%)	
Birth weight ≤2.5 kg	13	65	9	40.90	0.314
Nursery admission	1	5	3	13.63	0.782
Neonatal mortality	1	5	0	-	0.658
Stillbirth	6	30	2	9.09	0.309

The time between birth and the start of the fit in this trial also significantly influences the maternal outcome. Females with >24 hours between birth and the beginning of fits had an increased risk of developing HELLP syndrome, disseminated intravascular coagulation, acute renal failure, and PRES.

## Discussion

It was seen that in the present study, the incidence of eclampsia was found to be 0.34% of total deliveries studied. These findings were much lower and in contrast with the studies from Tanzania, Nigeria, Eastern India, Uttar Pradesh, and Karnataka, where the incidence was reported to be 1.37%, 7.8%, 3.2%, 2.2%, and 1.82% by Ndaboine et al. [8] in 2012, Efetie et al. [9] in 2007, Kumar et al. [10] in 2014 and Yaliwal et al. [11] in 2011.

The fact that the majority of the subjects in the institute under consideration in the current study are from a single geographic area and that only a small portion are complicated cases referred from other institutes can be used to explain the difference in preeclampsia incidence reported between the two studies. Additionally, as stated by Zwart et al. [12] in 2008 and Lal et al. [13] in 2013, the incidence recorded in industrialized nations is significantly lower as a result of simple and widespread access to the healthcare system and the acceptance of prenatal care by all pregnant women. 

The mortality and morbidity rates seen in the eclamptic participants in the current research were comparable to those found in studies from other parts of India and other developing nations. In the current investigation, a mortality rate of 9.52% (n=4) was noted. The fatality rates reported by Edgar et al. [8] in 2012 and Onhu et al. [14] in 2004 were 10.7% and 7.89%, respectively, in studies from Benin and Tanzania. These findings were comparable to those studies. 

These death rates were much higher than those of affluent nations, where Douglas et al. [15] reported a rate of 0.5% to 1.8% in 1994, and somewhat higher than those of East India, where Singh and Behera [16] recorded a rate of 4.4% in 2011. This discrepancy may be caused by a variety of factors, including poor transportation services, delays in referring the patient, increased travel time to medical facilities, and the subject's serious state upon arriving at the institute. The four fatalities described in the current investigation all had preventable contributing causes. Three women received no prenatal treatment. While being transported to the institution, two women experienced recurring convulsions, and at the medical facilities they visited, two females did not receive magnesium sulphate which was similar to the information provided by the ACOG practice bulletin [2].

It was noticed that only 42.85% (n=18) of the study participants visited a doctor prior to the commencement of convulsions, while 57.14% (n=24) did not get prenatal care at all. Prior to becoming pregnant, high blood pressure was noted in 4.76% (n=2) study females, although proteinuria was only noted in 2.38% (n=1) research females. It was discovered that only six out of 18 females who visited a healthcare facility for prenatal care had their blood pressure and proteinuria assessed. 

The study shows that poor results are typically linked to the subpar prenatal care given to women and their severe condition upon arrival. These results were in line with those of studies by Katz et al. [17] and Ray et al. [18] from 2000 and 2001, respectively, where the authors took the same variables into account for poor eclampsia outcomes.

The mortality and morbidity of eclampsia in females were substantially controlled by the incidence of complications. The presence of these problems presents a number of treatment issues for eclamptic people that enhance the results. The current study indicated a death rate of 22%. These death rates were comparable to those reported in other studies by Edgar et al. [8], Onhu et al. [14], Singh and Behera [16], and from 2012, 2004, and 2011 respectively.

According to Ray et al. [18] and Chappell et al. in 2001, women with eclampsia often have a higher risk of being small for gestational age and premature newly born [19]. The authors' findings and those of the current investigation were comparable. The authors identify a number of variables, such as multiple comorbidities disorders, delivery to commencement of fit interval, and delay in referral, as potential causes of the high observed rates of perinatal death. The findings of this study showed that asphyxia and low birth weight were the primary causes of perinatal death in the majority of cases. Due to the increased prevalence of preterm births in participants with eclampsia, the low birth weight number was much greater in the current research.

Limitations

The study had a few limitations, including the study being conducted in a single or limited geographical area, including the females visiting a single institute for delivery, all the subjects were not followed for complete pregnancy duration, and a smaller sample size of the study. The records used in this study were not present for research purposes.

## Conclusions

Most of the research participant women, had intrapartum eclampsia, postpartum eclampsia, and antepartum eclampsia, based on the time of the convulsions in relation to the labor. It was highlighted that there was no conclusive evidence linking the date of the fit's beginning to unfavourable results or an elevated risk of complications. Neonatal mortality and stillbirth were observed with vaginal delivery in eclampsia cases.

The conclusion drawn from the present study is that outcomes in eclampsia can be improved by termination of eclamptic subjects, early treatment initiation, timely and appropriate referral, early disease recognition, and appropriate antenatal care. The subjects with eclampsia should be treated at centres with a multidisciplinary approach, NICU (neonatal ICU), and ICU (intensive care unit) facilities. 

This study puts forward a few recommendations. Various steps should be taken to control blood pressure during pregnancy, including lifestyle changes, diet modification, and exercises during the pregnancy period. Also, effective and appropriate management techniques should be included in the curriculum of medical students and continuing medical education shall be conducted from time to time to get updates regarding eclampsia and its management.
